# *Staphylococcus aureus* Nasal Carriage among Beefpacking Workers in a Midwestern United States Slaughterhouse

**DOI:** 10.1371/journal.pone.0148789

**Published:** 2016-02-11

**Authors:** Jessica H. Leibler, Jeanne A. Jordan, Kirsten Brownstein, Lina Lander, Lance B. Price, Melissa J. Perry

**Affiliations:** 1 Department of Environmental Health, Boston University School of Public Health, Boston, Massachusetts, United States of America; 2 Department of Epidemiology and Biostatistics, Milken Institute School of Public Health, The George Washington University, Washington, District of Columbia, United States of America; 3 Department of Environmental and Occupational Health, Milken Institute School of Public Health, The George Washington University, Washington, District of Columbia, United States of America; 4 Department of Epidemiology, College of Public Health, University of Nebraska Medical Center, Omaha, Nebraska, United States of America; 5 Division of Pathogen Genomics, the Translational Genomics Research Institute, Flagstaff, Arizona, United States of America; University Hospital Münster, GERMANY

## Abstract

Occupational contact with livestock is an established risk factor for exposure to livestock-associated methicillin-resistant *Staphylococcus aureus* (MRSA), particularly among industrial swine workers. While *S*. *aureus* is known to infect cattle, livestock-associated *S*. *aureus* carriage among workers in the beef production chain has received limited attention. Beefpacking workers, who slaughter, butcher and process cattle, have intensified exposure to potentially infectious animal materials and may be at risk of livestock-associated *S*. *aureus* exposure. We conducted a cross-sectional study of beefpacking workers (n = 137) at an industrial slaughterhouse in the Midwestern United States to evaluate prevalence and characteristics of *S*. *aureus* nasal colonization, specifically the absence of the *scn* gene to identify putative association with livestock, antibiotic susceptibility, presence of Panton-Valentin leukocidin (PVL) genes *lukS*-PV and *lukF*-PV, and *spa* type. Overall prevalence of *S*. *aureus* nasal carriage was 27.0%. No workers carried livestock-associated MRSA. Methicillin-sensitive *S*. *aureus* isolates (MSSA) recovered from five workers (3.6%) lacked the *scn* gene and were considered putative livestock-associated *S*. *aureus* (pLA-SA). Among pLA-SA isolates, *spa* types t338, t748, t1476 and t2379 were identified. To our knowledge, these *spa* types have not previously been identified as associated with livestock. Prevalence of human-adapted MRSA carriage in workers was 3.6%. MRSA isolates were identified as *spa* types t002, t008 and t024, and four of five MRSA isolates were PVL-positive. To date, this is the first study to indicate that industrial beefpacking workers in the United States may be exposed to livestock-associated *S*. *aureus*, notably MSSA, and to *spa* types not previously identified in livestock and livestock workers. Occupational exposure to livestock-associated *S*. *aureus* in the beef production chain requires further epidemiologic investigation.

## Introduction

*Staphylococcus aureus* is known to colonize many livestock species, including swine, poultry, sheep and cattle [1–3]. Since 2003, nasal colonization with methicillin-resistant *S*. *aureus* (MRSA) ST398 has been documented frequently in livestock and persons who work with livestock [4–7], with emerging reports of clinical infection [8]. Nasal carriage is an epidemiologic biomarker of *S*. *aureus* exposure associated with increased risk of infection [9–11], and is used widely in research to assess human exposure to livestock-associated MRSA (LA-MRSA) [4]. While much of the research on LA-MRSA focuses on nasal carriage among swine workers, less is known about *S*. *aureus* colonization among workers in the beef production chain. *S*. *aureus* is a documented cause of bovine mastitis among dairy cattle [12–14], and recent reports from Europe indicate that cattle may be a reservoir of an emerging strain of MRSA infecting humans [15,16].

MRSA carriage has been documented in beef cattle, veal calves, and dairy cattle, and MRSA isolates have been recovered in bulk milk and consumer beef products [12,17–22]. MRSA surveillance studies of pre-slaughter beef cattle populations reflect evidence of regional variation and differences in prevalence along stages in the beef production chain. Four studies in the Netherlands identified prevalence of MRSA nasal carriage ranging from 3.9% to 24.8% among beef cattle in that country, with elevated prevalence noted among veal calves compared to beef and dairy cattle [21,23–25]. Researchers identified 8.7% prevalence of MRSA among nasal swabs from beef cattle in Germany [26]. However, Weese and colleagues did not find evidence of MRSA nasal carriage among beef cattle at feedlots in Canada, suggesting regional variability in MRSA presence in cattle [27]. In studies of *S*. *aureus* carriage among beef cattle, the majority of isolates have been identified as ST398, mostly *spa* types t011 and t034, although *spa* types not associated with ST398 have been recovered from beef and carcasses, including t1430 and t008 [26].

Among livestock farmers, occupational contact with cattle is a risk factor for nasal colonization with ST398 [16,17,28–31]. Intensity of animal contact has been identified as a risk factor for nasal carriage of LA-MRSA among workers [24,32] as has MRSA contamination in the occupational environment [33]. Meatpacking and slaughterhouse workers experience occupational exposure to potentially infectious animal material, however to date, only a limited number of studies have evaluated livestock-associated *S*. *aureus* (LA-SA) exposure in this workforce [34–38], all of which have studied swine or poultry workers. Animal slaughter and meatpacking workers in the United States have among the highest rates of occupational injury among all industries, notably laceration injuries, which pose exposure pathways for bacteria that may enter the body through breaks in the skin, such as *S*. *aureus* [39–41]. To our knowledge, no prior studies have evaluated *S*. *aureus* carriage among beef slaughterhouse workers in the United States.

We report here on a study of *S*. *aureus* nasal carriage among beefpacking workers employed at an industrial slaughter and meatpacking facility in the United States. The intentions of this study were to evaluate prevalence and characteristics of *S*. *aureus* nasal colonization in a sample of United States industrial beefpacking workers, specifically livestock-association, antibiotic susceptibility, *spa* type and presence of the Panton-Valentin leukocidin (PVL) genes *lukS*-PV and *lukF*-PV, to inform livestock-associated *S*. *aureus* exposure risk in this industry.

## Materials and Methods

### Study design and conduct

We conducted a cross-sectional study in June 2012 in small town in eastern Nebraska, United States, where an industrial beefpacking plant that employed approximately 2000 unionized workers was located. Individuals were eligible to participate in the study if they currently worked at the plant, spoke English or Spanish, were 18 years or older and had not traveled outside the country in the past three months. Workers were recruited by union leadership within the plant and by word of mouth to result in a sample representing employees working in the kill or cut floor of the plant, the two largest departments. Convenience sampling was used within these departments to recruit participants.

All aspects of the design and conduct of the study were approved by the Institutional Review Board at George Washington University. Participants were enrolled at a local union hall over a four-day period and all participants provided written informed consent. Trained study personnel conducted 30-minute interviews with each participant and collected nares swab samples to assess *S*. *aureus* nasal carriage. BBL Culture Swab Plus collection and transport device containing Amies gel without charcoal (Cat. No. 220116, Becton Dickinson, Franklin Park, MD) were used to collect nasal samples. The swab tip was inserted approximately 2 cm into the first naris along the medial aspect, and rotated slowly. The procedure was repeated in the second naris, and then the swab was returned to the transport sleeve, labeled, and placed in a refrigerated container on site. Participants were queried on work practices and behaviors, recent medical history, recent workplace injuries and demographics using a standard instrument that was validated in prior studies of meatpacking workers. Participants received $20 for joining the study.

### Laboratory analyses

At the end of each enrollment day, nares swabs were shipped overnight on cold packs to Dr. Jordan’s research laboratory at the George Washington University. Upon receipt, swabs were placed into BBL Trypticase Soy Broth with 6.5% sodium chloride (Cat. No. 221351) and incubated overnight at 37°C before being plated onto BD BBL CHROMagar MRSA II plates (Cat. No. 215228) and BBL Trypticase Soy Agar with 5% sheep blood (Cat. No. 221239) and again incubated overnight at 37°C. The resulting culture plates were inspected for growth. Colonies with characteristic morphology for *Staphylococci* found to be catalase positive, coagulase positive, Gram-positive cocci in clusters were considered presumptive *S*. *aureus* isolates and were screened for mecA, SCCmec and staphylococcal protein A genes to confirm MRSA using the Xpert® SA Nasal Complete assay (Catalog No. GXSACOMP-10, Cepheid, Sunnyvale, CA). *Spa* typing and real-time PCR for PVL (*lukS-PV* and *lukF-PV*) genes were conducted at the District of Columbia Department of Health. *Spa* types were determined through harmonization with Ridom *Spa*Server (http://spaserver.ridom.de). Isolates were also analyzed for the presence of the *scn* gene, a biomarker of human-association in *S*. *aureus*, at the Translational Genomics Research Institute (TGen) in Flagstaff, AZ per the approach developed by Stegger at al. [42] and used in other studies as a biomarker of livestock-association in *S*. *aureus* [43,44]. *Scn*-negative isolates were designated as putative livestock-associated *S*. *aureus* (pLA-SA). Confirmed *S*. *aureus* colonies were evaluated at TGen for susceptibility to 12 antimicrobials, representing seven classes of drugs, using Kirby-Bauer disk diffusion and interpreted per CLSI guidelines [45].

### Statistical analyses

Prevalence of *S*. *aureus*, MRSA, and pLA-SA nasal carriage was calculated, for the study population as a whole and for demographic (race/ethnicity, sex, age, educational level, household size) and occupational (job area in the plant and duration of employment) subgroups. Fisher’s exact tests were used to assess associations between pLA-SA and MRSA nasal carriage and demographic, employment and medical covariates. Wilcoxon rank sum tests were used to evaluate differences in number of antibiotics to which isolates were susceptible by demographic, employment and *S*. *aureus* categories.

## Results

### Study participants

During enrollment, 151 persons were recruited and interviewed at the union hall. Fourteen workers were excluded per study inclusion criteria, for non-English, non-Spanish speaking (n = 10), recent travel (n = 3) and not actively working at the plant (n = 1). In total, 137 participants met inclusion criteria and were enrolled in the study. Ninety-two percent of study participants identified as Latino or Hispanic, and mean age was 44 years (Table 1). Of study participants 55.5% were men (n = 76) and 44.5% were women (n = 61). The majority of study participants (55%; n = 74) had worked in the plant for five or more years. Approximately 50% of participants worked on the cut floor, cutting and butchering animals for packaging, while 30% worked on the kill floor, slaughtering animals and hanging and preparing carcasses, which represented the overall distribution of employment within the plant (Table 1).

**Table 1:** Demographic and employment characteristics of beef meatpacking workers, Nebraska, United States (n = 137).

Characteristic^a^	Number of workers (%)
**Sex**	
Male	76 (55.5)
Female	61 (44.5)
**Race/ethnicity**	
White/Caucasian	10 (7.3)
Latino/Hispanic	126 (92.0)
Other	1 (<1)
**Current smoking status**	
Non-smoker	118 (86.7)
Smoker	18 (13.2)
**Education**	
Less than high school	75 (54.7)
High school or more	62 (45.3)
**Duration of employment at this plant**	
<1 year	17 (12.4)
1–5 years	46 (33.6)
5–10 years	27 (19.7)
10+ years	47 (34.3)
**Department/work area**	
Kill floor	40 (29.2)
Cut floor	71 (51.8)
Box room	8 (5.8)
Shipping	5 (3.6)
Other	13 (9.5)

^a^ Mean age of workers was 44 years (standard deviation: 11 years)

### *S*. *aureus* nasal colonization

Prevalence of *S*. *aureus* nasal carriage was 27.0% (37 of 137) (Table 2; S1 Data). Five *S*. *aureus* isolates lacked the *scn* gene and therefore were identified as having putative association with livestock (3.6% of all study participants; 13.5% of *S*. *aureus* carriers) (Table 2). All of the pLA-SA isolates were identified as MSSA. Of all MSSA isolates, 15.6% were pLA-SA.

**Table 2:** Prevalence of *Staphylococcus aureus* nasal carriage, US beefpacking workers (n = 137).

Characteristic	Prevalence % (n)	Prevalence ratio for *S*. *aureus*^b^	Multidrug resistant *S*. *aureus* % (n)^c^	PVL-positive % (n)^d^
***Staphylococcus aureus* (all)**	27.0 (37/137)	-	10.2 (14/137) of all subjects; 38.9 (14/37) of *S*. *aureus* carriers	2.9 (4/137)
Methicillin-sensitive *S*. *aureus* (MSSA)	23.3 (32/137)	86.5	28.1 (9/32)	0 (0/5)
Methicillin-resistant *S*. *aureus* (MRSA)	3.6 (5/137)	13.5	100 (5/5)	80.0 (4/5)
Putative livestock-associated *S*. *aureus* (pLA-SA)^a^	3.6 (5/137)	13.5	40.0 (2/5)	0 (0/5)

^a^ Defined as all *scn*-negative *S*. *aureus;* all pLA-SA isolates were MSSA

^b^ Prevalence ratio defined as: # *S*. *aureus* isolates in specific category/all *S*. *aureus* positive isolates (n = 37)

^c^ Defined as resistant to ≥3 antibiotics tested

^d^ Positive for the *lukS-PV* and *lukF-PV* genes by real-time PCR

No workers carried livestock-associated MRSA; all recovered MRSA isolates were *scn*-positive, indicating human adaptation. Prevalence of MRSA nasal carriage among study participants was 3.6% (5 of 137). MRSA accounted for 13.5% of all *S*. *aureus* isolates.

#### *Spa* typing

*Spa* types among pLA-SA isolates did not cluster and no duplicates were observed (Table 3). *Spa* types identified among *scn*-negative isolates were t338, t748, t1476 and t2379 (Table 3). One *scn*-negative isolate was non-typeable.

**Table 3:** *Spa* types and genetic characteristics of select *S*. *aureus* isolates recovered from US beefpacking workers (n = 37 carriers)^a^.

Ridom *spa* type^c^	Number (n = 37)	*scn*-negative^b^ (n = 5)	PVL-positive (n = 4)	MRSA (n = 5)
t002	2 (5.4)	0	0	1 (20)
t008	2 (5.4)	0	2 (50)	2 (40)
t024	2 (5.4)	0	2 (50)	2 (40)
t338	1 (2.7)	1 (20)	0	0
t748	1 (2.7)	1 (20)	0	0
t1248	4 (10.8)	0	0	0
t1476	1 (2.7)	1 (20)	0	0
t2379	1 (2.7)	1 (20)	0	0

^a^ Five MSSA isolates were non-typeable, including one *scn*-negative isolate.

^b^ *scn* negative defined as presumptive livestock-associated *S*. *aureus*. One *scn*-negative isolate was non-typeable.

^c^ The following 15 *spa* types were recovered from one worker each (n = 1; 2.7%), were all *scn*-positive, PVL negative and MSSA: t021, t065, t078, t189, t276, t304, t458, t571, t688, t701, t992, t1250, t3182, t4298, t4976, t6150.

Among all *S*. *aureus* isolates, the largest *spa* cluster observed was type t1248, all of which were MSSA (n = 4; 10.8% of *S*. *aureus* isolates) (Table 3). A cluster of *spa* type t065 (n = 3; 8.1% of *S*. *aureus* isolates) was identified; these isolates were also MSSA. The MRSA isolates were associated with three *spa* types: t002, t008 and t024. MRSA isolates clustered in two *spa* types: t008 (40% of MRSA isolates) and t024 (40% of MRSA isolates). Two isolates were identified as *spa* type t002, one of which was MRSA and the other MSSA. All other *S*. *aureus* isolates represented distinct *spa* types (t021, t065, t078, t189, t276, t304, t458, t571, t688, t701, t992, t1250, t3182, t4298, t4976, t6150). Of all the *S*. *aureus* isolates, five (3.6%) were non-typeable. There was no overlap between the *spa* types observed in the livestock-associated and human-associated isolates.

#### Panton-Valentin leukocidin genes

Four isolates were PVL-positive, all of which were MRSA and not associated with livestock (Table 3). None of the pLA-SA or MSSA isolates contained the *lukS-PV* and *lukF-PV* genes.

#### Antibiotic susceptibility

Of the five pLA-SA isolates, all were resistant to penicillin and four (80%) were resistant to ampicillin (Table 4). Two pLA-SA isolates were resistant to both erythromycin and clindamycin (using the D-Test for inducible clindamycin resistance) and one isolate was resistant to tetracycline. Greater multidrug resistance (resistant to ≥3 antibiotics) was observed among the pLA-SA isolates compared to the human associated isolates, but this finding was not statistically significant at the 5% level. We observed suggestive elevated prevalence of tetracycline resistance among the pLA-SA isolates compared to the human-adapted isolates (20% vs. 3.2%), but these findings were not statistically significant (Table 4).

**Table 4:** Prevalence of antibiotic resistance (% resistant) among *S*. *aureus* isolates recovered from nasal swabs of US beefpacking workers (n = 37 *S*. *aureus* carriers).

% Isolates resistant to antibiotic					
Antibiotic name ^a^	All *S*. *aureus*	MSSA^b^	MRSA^c^	Putative livestock-associated *S*. *aureus* (pLA-SA)^d^	Putative human-adapted *S*. *aureus*)^e^
Levofloxicin	2.8	0	20.0	0	3.2
Ciprofloxicin	2.8	0	20.0	0	3.2
Tetracycline	5.6	6.5	0	20.0	3.2
Ceftriaxone	13.9	0	100	0	16.1
Clindamycin^f^	25.0	25.8	20.0	40.0	22.6
Erythromycin	33.3	29.0	60.0	40.0	32.3
Ampicillin	72.2	67.7	100	60.0	74.2
Penicillin	80.6	77.4	100	80.0	80.7

^a^All recovered isolates were sensitive to the following antibiotics: rifampin, linezolid, quinupristin/dalfopristin, gentamicin, and trimethoprim/sulfamethoxazole

^b^Methicillin-sensitive *S*. *aureus*

^c^Methicillin-resistant *S*. *aureus*

^d^Putative livestock-associated *S*. *aureus* (pLA-SA) demonstrated by lack of *scn* gene

^e^Putative human-associated *S*. *aureus* demonstrated by presence of *scn* gene

^f^D-test for inducible clindamycin resistance used to evaluate clindamycin resistance

One MRSA isolate was resistant to eight individual antibiotics tested, including levofloxacin, ciprofloxacin, erythromycin, and clindamycin. All MRSA isolates were susceptible to tetracycline, rifampin, linezolid, quinupristin-dalfopristin, gentamicin, and trimethoprim-sulfamethoxazole (Table 4).

MRSA isolates were resistant to a greater number of antibiotics tested compared to MSSA isolates (p = 0.005). All of the MRSA isolates were multidrug resistant, compared to 29.0% of the MSSA isolates (Table 2). All *S*. *aureus* isolates recovered were susceptible to rifampin, linezolid, quinupristin-dalfopristin, gentamicin, and trimethoprim-sulfamethoxazole (Table 4).

### Risk factors for nasal colonization

We observed no association between recent occupational injury, infection, duration of employment, or demographic factors and pLA-SA nasal carriage. There was no association between work area (kill or cut floor) or identified occupational activities or behaviors and risk of pLA-SA carriage.

Workers with larger households (> 6 persons) were marginally more likely to be MRSA nasal carriers compared to workers with smaller households (p = 0.08). Workers who reported having household members who also worked in meatpacking were also marginally more likely to have MRSA nasal carriage compared to workers without household members employed in this industry (p = 0.06). Neither association was significant in regard to pLA-SA colonization.

## Discussion

In total, five workers in our study (3.6%) were nasal carriers MSSA with putative association with livestock. These pLA-SA isolates represented distinct *spa* types (t338, t748, t1476, t2379) that are associated with strains not previously identified in livestock, to our knowledge. *Spa* type t338 is associated with ST30, a worldwide epidemic *S*. *aureus* clone that is frequently PVL positive, although the isolate recovered in this study was PVL negative. *Spa* type t1476 is associated with ST-8, a MSSA strain recovered in the United States and Europe. The other *spa* types identified as livestock-associated (t748 and t2379) are associated with *S*. *aureus* strains recovered with low frequency in Europe. It is possible that these strains have a previously unidentified association with livestock, or that our findings reflect subclinical or background circulation of MSSA strains in the United States that are not specific to beefpacking workers. A larger epidemiologic study that surveys both beefpacking workers and community members who do not work in this industry would inform the meaning of this observation, and is recommended. Studies that sample livestock for *S*. *aureus* during production and slaughter, as well as environmental sampling of the slaughterhouse facility, would also inform worker exposures.

We observed no indication of worker carriage with *S*. *aureus spa* types associated with ST398, the MRSA strain identified widely in swine, cattle, poultry and individuals with direct contact with livestock. MRSA isolates recovered from beefpacking workers in this study were human adapted, community-acquired strains. The MRSA *spa* types identified from our study participants reflected MRSA clonal complexes with epidemic distribution, including CC5, USA100 and eMRSA-3 (t002), CC8 and USA300 (t008) and ST-8 (t024). Of note, CC8 has been identified in cattle in Europe in recent years [21], although the isolate of *spa* type t008 in our study was human, not livestock-adapted.

A recent study of MRSA-infected patients at a rural hospital in Pennsylvania by Casey and colleagues indicates that other *S*. *aureus* strains with livestock association, in addition to ST398, may infect humans in areas with intensified exposure to livestock production [46]. Although we did not observe LA-MRSA in our study, our findings reinforce the conclusion that a singular focus on ST398 as the marker of livestock association may be too narrow. Other recent studies indicate a broad diversity of MSSA within the agricultural environment, and our findings support these observations [47].

Although not statistically significant, prevalence of tetracycline resistance was elevated in the livestock-associated isolates compared to the human-adapted isolates. Tetracycline is intensively used in animal agriculture and resistance to it is commonly identified in livestock-associated *S*. *aureus* isolates [48]. Our findings reinforce previous work that identifies tetracycline resistance as a marker of livestock-association in *S*. *aureus*.

Prevalence of MRSA nasal carriage among this sample of US beef meatpacking workers was three times higher than general population estimates in the United States, which is approximately 1.2% [49]. While overall prevalence of *S*. *aureus* nasal carriage in our study paralleled national estimates, the proportion of *S*. *aureus* isolates that were MRSA in our study (13.5%) was notably higher than the proportion of MRSA to all *S*. *aureus* isolates observed in the general population (3.9%) [49]. This finding indicates a potentially higher risk among this workforce for infections associated with *S*. *aureus* carriage compared to the general population.

The elevated prevalence of MRSA nasal carriage among this workforce may reflect increased risk of human-to-human transmission within the workplace or household. Household transmission is an important source of spread of MRSA within families, and larger households pose a greater risk of pathogen transmission within the home [50]. Workers in our study reported varying household sizes, ranging from 1–11 persons, with the median household size of four persons, and we observed a suggestive association between household size greater than six persons and MRSA nasal carriage. Overcrowded housing conditions are common among immigrant agricultural workers, and crowded living conditions have been documented to increased risk of disease transmission in this population [51]. While our study was not designed to evaluate specifics of the relationship between housing characteristics and infection among this workforce, it is an important area for future study.

Our study was limited by small sample size and potential for selection bias in enrollment. While our sample did achieve a representative sample of workers in the two main work areas, the kill and cut floors, we were constrained in our ability to recruit a sizable proportion of the workforce due to budgetary limitations. Our small sample size may have reduced our ability to draw firm conclusion regarding risk factors for pLA-SA nasal carriage, including occupational activities and behaviors. Despite the limitations inherent in small sample size, potential participants were blinded to the specific nature of the study during recruitment, so a biased sample in regard to our outcome of prevalence of pLA-SA nasal carriage was unlikely. Our sample also paralleled overall employee demographics in the plant, in regard to race/ethnicity, sex and age, suggesting the participants were representative of the overall plant workforce. We were also unable to recruit workers in departments without animal contact in a meaningful way due to the pilot nature of this study, which did not allow us to evaluate carriage in these workers. A larger study that recruits more workers at multiple beefpacking plants, including those in jobs in the plant without animal contact (such as administration or packaging), would be valuable to further inform LA-SA carriage in this workforce.

To our knowledge, this is the first study to evaluate livestock-associated *S*. *aureus* carriage among beefpacking workers in the United States. Our findings indicate that beefpacking workers in one plant are colonized with pLA-SA of *spa* types not previously associated with livestock, although specific occupational practices related to exposure are unclear. Workers in this plant also experienced elevated prevalence of MRSA nasal carriage compared to general population estimates, which may be mediated by household crowding. Future studies are needed to elucidate occupational exposure to *S*. *aureus* among industrial beefpacking workers and to inform occupational practices that increase risk of pLA-SA exposure.

## Supporting Information

S1 Data (XLSX)
